# Factors Associated With Antiseizure Medication Adherence in Patients With Epilepsy: A Systematic Review

**DOI:** 10.1002/brb3.71550

**Published:** 2026-06-14

**Authors:** Gülcan Bahçecioğlu Turan, Mizgin Demir

**Affiliations:** ^1^ Nursing Department, Faculty of Health Sciences Fırat University Elazığ Türkiye; ^2^ Nursing Department Faculty of Health Sciences Fırat University Elazığ Türkiye

**Keywords:** antiseizure medication, epilepsy, medication adherence, systematic review, treatment adherence

## Abstract

**Introduction:**

Epilepsy is one of the most common chronic neurological disorders worldwide, and adherence to antiseizure medication (ASM) therapy is essential for seizure control and improved quality of life. Poor adherence has been associated with increased morbidity, mortality, and healthcare burden. This systematic review aimed to examine factors associated with ASM adherence among patients with epilepsy across different sociocultural and clinical settings.

**Methods:**

Systematic searches were conducted in PubMed, Scopus, Web of Science, and Google Scholar databases. A total of 5736 records were identified and screened according to the PRISMA 2020 guidelines. After applying predefined eligibility criteria and assessing methodological quality using the Joanna Briggs Institute (JBI) Critical Appraisal Checklists, 41 quantitative studies were included in the final synthesis.

**Results:**

Most studies reported moderate levels of ASM adherence. Factors associated with nonadherence were categorized according to the five World Health Organization (WHO) dimensions of adherence. The most frequently reported barriers included forgetfulness, complex medication regimens (particularly polytherapy), and adverse drug effects. Psychosocial factors such as depression, anxiety, negative medication beliefs, and perceived stigma were consistently associated with lower adherence. In addition, socioeconomic barriers including low income and high medication costs were frequently associated with poor adherence, particularly in low‐ and middle‐income countries.

**Conclusion:**

ASM adherence in epilepsy is associated with a complex interaction of biological, psychosocial, cultural, and economic factors. Major barriers include forgetfulness, treatment complexity, adverse effects, mental health problems, stigma, and financial difficulties. Improving adherence may require multidimensional interventions, including individualized patient education, psychosocial support, simplified treatment regimens, and strengthened healthcare policies targeting both patient‐ and system‐level barriers.

## Graphical abstract

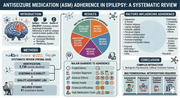

## 1 Introduction

Epilepsy is a heterogeneous chronic neurological disorder characterized by various syndromic types, diverse etiologies, and variable prognoses (Dima and Shibeshi 2022; Manole et al. 2023). Affecting approximately 50 million people worldwide, it is considered a major global public health concern, with a prevalence ranging from 4 to 10 per 1000 individuals (Gurumurthy et al. 2017; WHO 2024). The primary goals in epilepsy management are to eliminate or reduce the frequency of seizures, minimize treatment‐related side effects, and maintain the patient's psychosocial functioning (Akoluk and Ovayolu 2021; Narducci et al. 2023).

Studies indicate that over 70% of patients can achieve seizure control when antiseizure medications (ASMs) are taken regularly (Endayen et al. 2023; Panayiotopoulos 2005). This highlights the central role of treatment adherence in epilepsy management. Treatment adherence refers to the extent to which a patient's medication‐taking behavior aligns with the prescribed therapeutic regimen (Hasiso and Desse 2016). However, a significant proportion of individuals with epilepsy fail to achieve seizure control despite appropriate therapeutic regimens, with the most common reason being irregular or inadequate medication use (Hasiso and Desse 2016; Ferrari et al. 2013).

Importantly, medication adherence carries uniquely critical implications in epilepsy compared to other chronic diseases. ASMs have extremely low drug forgiveness, meaning that even short delays or missed doses can rapidly lead to breakthrough seizures (Chen et al. 2010; Donahue et al. 2025). Unlike many chronic therapies in which occasional nonadherence has minimal short‐term clinical consequences, the narrow therapeutic window and required stable serum levels of ASMs make epileptic patients highly vulnerable to missed doses (Josephson et al. 2017). This pharmacological sensitivity increases the risk of sudden seizure recurrence, injuries, hospitalization, and significant declines in quality of life (Bayane and Senbeta 2023). Therefore, understanding the multifaceted factors influencing adherence in epilepsy is essential not only for optimizing long‐term disease control but also for preventing preventable morbidity and acute complications.

Poor adherence contributes to persistent seizures, increased emergency visits, reduced quality of life, and elevated mortality risk (Narducci et al. 2023; Solomon et al. 2023). Adherence is a multifactorial issue influenced not only by individual motivation but also by socioeconomic conditions, healthcare system structures, and disease‐ or treatment‐specific factors. The World Health Organization (WHO) categorizes these influences into five key dimensions: social and economic factors, therapy‐related factors, patient‐related factors, condition‐related factors, and healthcare system–related factors (Sabaté 2003). Recent analyses, including Kardas et al. (2024), emphasize that medication nonadherence remains a major global health challenge even two decades after the WHO report, underscoring the continued relevance of this framework (Kardas et al. 2024). In epilepsy, specific barriers include ASMs side effects, disease duration and severity, individual limitations, and lack of knowledge (Getnet et al. 2016; Verma et al. 2018). Psychological factors also play a critical role; for instance, internal resources such as high self‐esteem may enhance adherence (Motioleslam et al. 2024). Social influences, including patients’ tendency to hide their illness or perceive it as shameful, can further negatively affect adherence (Akoluk and Ovayolu 2021; Chesaniuk et al. 2014).

This study aims to systematically identify and synthesize the biological, psychological, treatment‐related, disease‐related, socioeconomic, cultural, and health system factors associated with medication adherence in individuals with epilepsy, based on observational studies published in the last 15 years. This review specifically maps these factors onto the five WHO dimensions of medication adherence in order to provide a structured, theory‐driven understanding of the determinants of adherence in epilepsy.

## 2 Methods

### 2.1 Study Design

This review was conducted as a systematic review rather than a scoping review because the aim was to critically appraise and synthesize existing evidence, assess methodological quality, and draw evidence‐based conclusions regarding factors influencing medication adherence. A scoping review would only map the literature without offering quality assessment or comparative synthesis, which did not align with the objectives of this study. This systematic review was carried out following the PRISMA (Preferred Reporting Items for Systematic Reviews and Meta‐Analyses) guidelines. This study is registered with PROSPERO (CRD420251036817). As the review involves analysis of previously published studies with anonymized patient data, ethical approval was not required.

### 2.2 Research Question Framework

This systematic review was structured according to the PICO framework, which is recommended for evidence synthesis in systematic reviews. The PICO model was selected because the purpose of this review was to identify factors associated with medication adherence and to compare adherence‐related characteristics across different patient groups, rather than to broadly map the literature as in scoping reviews.

- Population (P): Individuals diagnosed with epilepsy.
- Intervention/exposure (I): Factors influencing medication adherence.
- Comparator (C): Differences in adherence across demographic, clinical, psychosocial, or treatment‐related subgroups.
- Outcomes (O): Medication adherence levels and determinants of nonadherence.

Using the PICO framework allowed for a clear definition of the research focus, consistent eligibility criteria, and a structured synthesis aligned with systematic review methodology.

### 2.3 Eligibility Criteria

Inclusion criteria:

- Original research studies examining treatment adherence or factors affecting adherence among patients with epilepsy.
- Studies focusing on adherence issues related to antiepileptic drug use.
- Studies targeting human populations.
- Articles published in English or Turkish.
- Publications from 2010 onward.
- Full‐text accessible publications.
- Descriptive, cross‐sectional, and cohort studies.
- Studies scoring ≥ 7 on the Joanna Briggs Institute (JBI) Critical Appraisal Checklists were included.

Exclusion criteria:

- Systematic reviews, qualitative and interventional studies, case reports, experimental studies, editorials, book chapters, and letters to the editor.
- Studies published in languages other than English or Turkish.
- Publications without full‐text availability.
- Studies scoring < 7 on JBI Critical Appraisal Checklists.

### 2.4 Information Sources and Search Strategy

To ensure a comprehensive and reproducible search process, Medical Subject Headings (MeSH) terms and their corresponding synonyms were used in PubMed, while equivalent controlled vocabulary and keyword combinations were applied for Scopus and Web of Science.

The main MeSH terms included “Epilepsy” [MeSH], “Medication Adherence” [MeSH], and “Anticonvulsants” [MeSH]. These were combined with free‐text keywords such as “seizure disorder,” “treatment adherence,” “medication compliance,” “nonadherence,” “antiepileptic drugs,” “antiseizure medications (ASMs),” “predictors,” “determinants,” “barriers,” and “facilitators.” Boolean operators (AND/OR) were used to maximize search sensitivity and ensure inclusion of all relevant studies.

The literature search was conducted sequentially across databases using 20‐day structured intervals to ensure systematic coverage. PubMed was searched between January 1‐20, 2025, followed by Scopus (21 January–February 10, 2025), Web of Science (11 February–March 2, 2025), and Google Scholar (3 March–March 22, 2025). A detailed database‐specific search strategy is presented in Table 1. Google Scholar was also searched and yielded approximately 3000 records. Records were screened in order of relevance, starting from the most relevant results. Titles and abstracts were assessed for eligibility using predefined inclusion criteria. Screening was continued until no additional eligible studies were identified due to a progressive decline in relevance of retrieved records. Backward and forward citation tracking were performed. No gray literature sources were included. Duplicate records were removed using EndNote software, followed by manual verification.

**TABLE 1:** Literature search strategies in PubMed, Web of Science, Scopus, and Google Scholar.

Database	Search formula/strategy
PubMed	(“Epilepsy” [MeSH] OR epilepsy OR “seizure disorder”) AND (“Medication Adherence” [MeSH] OR “treatment adherence” OR “medication compliance” OR nonadherence) AND (“Anticonvulsants” [MeSH] OR “antiepileptic drugs” OR ASMs OR AEDs) AND (factors OR predictors OR barriers OR facilitators)
Web of Science	TS = ((epilepsy OR “seizure disorder”) AND (“treatment adherence” OR “medication compliance” OR nonadherence) AND (“antiepileptic drugs” OR ASMs) AND (factors OR predictors OR barriers OR facilitators))
Scopus	TITLE‐ABS‐KEY (epilepsy OR “seizure disorder”) AND TITLE‐ABS‐KEY (“treatment adherence” OR “medication compliance” OR nonadherence) AND TITLE‐ABS‐KEY (“antiepileptic drugs” OR ASMs OR AEDs) AND TITLE‐ABS‐KEY (factors OR predictors OR barriers OR facilitators)
Google Scholar	“epilepsy” AND (“treatment adherence” OR “medication compliance”) AND (“antiepileptic drugs” OR “antiseizure medications”) AND (factors OR predictors OR barriers)

### 2.5 Study Selection

**FIGURE 1:**
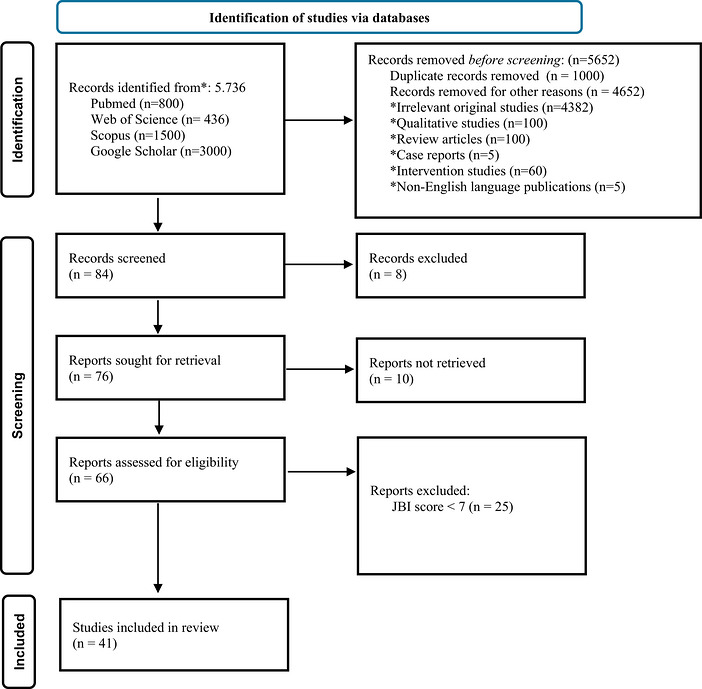
PRISMA 2020 flow diagram of the study selection process.

Two independent reviewers screened titles, abstracts, and full texts according to predefined eligibility criteria. Any disagreements were resolved through discussion and consensus. The study selection process is illustrated in the PRISMA 2020 flow diagram (Figure 1).

### 2.6 Data Extraction and Quality Assessment

Information from the selected studies was extracted using Microsoft Excel. Extracted data included study design, publication year, country, first author, study population, gender, age, sample size, adherence measurement tools, factors related to medication adherence, and key study outcomes. The methodological quality of the included studies was assessed using study design–specific JBI Critical Appraisal Checklists. Cross‐sectional studies were evaluated using the JBI Analytical Cross‐Sectional Checklist, and cohort studies were assessed using the JBI Cohort Checklist (Moola et al. 2020). Two independent reviewers performed data extraction and quality assessment. Any discrepancies were resolved through discussion and consensus. No third reviewer was involved. Studies scoring ≥ 7 on the JBI checklists were included in the review, while studies scoring < 7 were excluded during the eligibility assessment phase. This ensured that only methodologically sound studies were included in the final synthesis. The item‐level JBI critical appraisal results are presented in Table S1.

### 2.7 Mapping Factors to WHO Dimensions

A narrative thematic synthesis approach was used to analyze and integrate the findings of the included studies. The synthesis was guided by the WHO multidimensional adherence framework, which categorizes determinants of medication adherence into five domains: patient‐related, disease‐related, treatment‐related, social/economic, and health system‐related factors.

All extracted determinants were independently coded by two reviewers and mapped to the predefined WHO domains based on their conceptual relevance. In cases of disagreement, consensus was reached through discussion. When a factor overlapped multiple domains, it was assigned to the category representing its primary influence on medication adherence to ensure consistency. The direction of associations (positive or negative impact on adherence) was determined based on reported statistical analyses (e.g., odds ratios, regression coefficients, and *p*‐values) or, when unavailable, authors’ interpretations. Findings were synthesized narratively to identify recurring patterns across studies and to enable cross‐study comparison. Detailed domain‐level mapping of all determinants according to the WHO framework is presented in Table 3.

### 2.8 Risk of Bias

The methodological quality and risk of bias of the included studies were evaluated using the JBI Critical Appraisal Checklists appropriate for each study design. Cross‐sectional studies were assessed using the JBI Analytical Cross‐Sectional Checklist, while cohort studies were evaluated using the JBI Cohort Checklist. Two independent reviewers conducted all risk of bias assessments. Any disagreements were resolved through discussion and consensus. No third reviewer was required. Overall, the included studies demonstrated generally moderate to high methodological quality. Studies scoring below the predefined threshold (JBI < 7) were excluded during the eligibility assessment phase, ensuring that only studies with acceptable methodological rigor were included in the final synthesis. The detailed item‐level JBI appraisal results are provided in Table S1, while the overall risk of bias summary is presented in Table 2.

**TABLE 2:** Characteristics of included studies and risk of bias assessment.

First author of the study (year)	Study design	Country	Number of populations/participants	JBI score	Risk of Bias
Ferrari et al. (2013)	Prospective cross‐sectional study	Brazil	385 (18 years and older)	7	Low
Gurumurthy et al. (2017)	Cross‐sectional study	India	451 (18 years and older)	7	Low
Kumar et al. (2021)	Cross‐sectional study	India	268 (18 years and older)	7	Low
Korkmaz et al. (2020)	Cross‐sectional study	Türkiye	226 child and adolescent caregivers	7	Low
Dayapoğlu et al. (2021)	Descriptive, cross‐sectional	Türkiye	174 (18 years and older)	7	Low
Narducci et al. (2023)	Cross‐sectional study	Italy	200 (18 years and older)	7	Low
Guo et al. (2015)	Cross‐sectional study	China	184 (18 years and older)	7	Low
Verma et al. (2018)	Cross‐sectional study	India (rural)	230 (18 years and older)	7	Low
Abebe et al. (2022)	Cross‐sectional study	Ethiopia	187 (18 years and older)	7	Low
Dabilgou et al. (2025)	Cross‐sectional study	Burkina Faso	107 (18 years and older)	7	Low
Yang et al. (2023)	Cross‐sectional study	China	1847 children	7	Low
Choi et al. (2023)	Cross‐sectional study	New York, USA	644 (18 years and older)	7	Low
Dima and Shibeshi (2022)	Cross‐sectional study	Ethiopia	192 children	7	Low
Afsharkhas et al. (2022)	Cross‐sectional study	Iran	120 children	7	Low
Özer et al. 2023	Cross‐sectional study	Türkiye	150 (18 years and older)	7	Low
Fadare et al. (2018)	Cross‐sectional study	Nigeria	126 children	7	Low
Chauhan et al. (2018)	Cross‐sectional study	India	With 112 children and their caregivers	7	Low
Tan et al. (2015)	Cross‐sectional study	Malaysia	145 (18 years and older)	7	Low
Mamo et al. (2024)	Cross‐sectional study	Ethiopia	214	7	Low
Yang et al. (2020)	Cross‐sectional study	China	399 children	7	Low
Yang et al. (2025)	Cross‐sectional study	China	1357 children	7	Low
Hasiso and Desse (2016)	Cross‐sectional study	Ethiopia	194	7	Low
Akoluk and Ovayolu (2021)	Cross‐sectional study	Türkiye	325	7	Low
Asghar et al. (2021)	Cohort study	Pakistan	11,490	11	Low
Teh et al. (2020)	Cross‐sectional study	Malaysia	208	7	Low
Sunny et al. (2020)	Cross‐sectional study	India	304	7	Low
Solomon et al. (2023)	Cross‐sectional study	Ethiopia	352	7	Low
Motioleslam et al. (2024)	Cross‐sectional study	Iran	250	7	Low
Khatooni et al. (2024)	Cross‐sectional study	Iran	235	7	Low
Verma et al. (2020)	Cross‐sectional study	India	385	7	Low
Chen et al. (2010)	Cross‐sectional study	Taiwan	357	7	Low
Büyükbayram et al. (2023)	Cross‐sectional study	Türkiye	220	7	Low
Almwled et al. (2022)	Cross‐sectional study	Saudi Arabia	207	7	Low
Chesaniuk et al. (2014)	Cross‐sectional study	Colombia	140	7	Low
Liu (2013)	Cross‐sectional study	China	368 (all age groups)	7	Low
Getnet et al. (2016)	Cross‐sectional study	Ethiopia	450	7	Low
Niriayo et al. (2019)	Cross‐sectional study	Ethiopia	292	7	Low
Lin et al. (2018)	Cross‐sectional study	Iran	760	7	Low
Memiş and Koç (2020)	Cross‐sectional study	Türkiye	125 children and parents	7	Low
Suzuki et al. (2020)	Cross‐sectional study	Japan	855 (15 years and older)	7	Low
Taha et al. (2024)	Cohort	Scotland	266	9	Low

## 3 Results

### 3.1 Screening and Study Inclusion

A total of 5736 records were identified through database searches. After removing duplicates (*n* = 1000), 4736 records were screened. Of these, 4652 records were excluded during title and abstract screening due to irrelevance to the study topic and failure to meet the inclusion criteria. Full‐text assessment was conducted for 84 articles. Of these, 18 articles were excluded due to lack of full‐text availability. The remaining 66 full‐text articles were assessed for eligibility based on predefined inclusion criteria. Consequently, 41 studies met the inclusion criteria and were included in the final review (Figure 1).

### 3.2 Study Characteristics

Table 2 provides a detailed summary of the included studies, including country, study design, sample size, measurement tools, and main findings. The 41 included studies were conducted across Asia, Africa, North America, and Europe, with sample sizes ranging from 91 to 1357 participants. The majority employed cross‐sectional designs. The most commonly used adherence measurement tool was the Morisky Medication Adherence Scale (MMAS‐4/MMAS‐8), which allows quantitative comparison of adherence and nonadherence. Other frequently applied instruments included the Beliefs About Medicines Questionnaire (BMQ‐T), Beck Depression Inventory‐II (BDI‐II), DASS‐21, Generalized Anxiety Disorder‐7 (GAD‐7), Patient Health Questionnaire‐9 (PHQ‐9), Quality of Life in Epilepsy Inventory‐31 (QoLIE‐31), 14‐item Resilience Scale (RES14), and the 17‐item Public Health Literacy Knowledge Scale (PHLKS) (Dima and Shibeshi 2022; Narducci et al. 2023; Lin et al. 2018; Korkmaz et al. 2020).

### 3.3 Thematic Synthesis

Factors influencing medication adherence were categorized according to the five WHO dimensions: health system/team‐related, socioeconomic, therapy‐related, patient‐related, and condition‐related. Studies were conducted in countries including Brazil, India, China, Türkiye, Ethiopia, Scotland, Saudi Arabia, Iran, Italy, Japan, Nigeria, Malaysia, and the United States, revealing variability in adherence across different age groups and contexts. Nonetheless, most key determinants exhibited notable similarities across countries (Gurumurthy et al. 2017; Narducci et al. 2023; Ferrari et al. 2013; Dayapoğlu et al. 2021). A structured evidence synthesis of study characteristics, adherence measurement tools, adherence outcomes, and associated factors categorized according to WHO domains is presented in Table 3. This table provides a comprehensive overview of the included studies and supports cross‐study comparison of adherence determinants.

**TABLE 3:** The systematic review findings.

Study (year)	Country	Study design	Population	Gender	Adherence measure	Adherence level/prevalence	Key associated factors	WHO domain	Strength of association (OR/*p*‐value)
Ferrari et al. (2013)	Brazil	Prospective cross‐sectional study	Adults (*n* = 385)	206 women	Morisky–Green Test, Epilepsy Medication and Treatment Complexity Index (EMTCI).	66.2% nonadherence (moderate‐to‐low)	Male gender, young age, uncontrolled seizures, treatment complexity	Patient/disease/treatment	Female gender (*B* = −0.48, OR = 0.62, 95% CI: 0.39–0.97, *p* = 0.037), controlled seizure status (*B* = −0.56, OR = 0.57, 95% CI: 0.33–0.98, *p* = 0.041), EMTCI score (*B* = 0.61, OR = 1.06, 95% CI: 1.03–1.10, *p* < 0.001), and age (*B* = −0.27, OR = 0.97, 95% CI: 0.96–0.99, *p* = 0.003).
Gurumurthy et al. (2017)	India	Cross‐sectional study	Adults (*n* = 451)	251 men	4‐item Morisky (MMAS‐4)	72.3% high adherence; 27.7% low adherence	Socioeconomic status (SES), epilepsy type, forgetfulness	Social and economic/disease/patient	Middle/lower‐middle socioeconomic class (*B* = −0.660, OR = 0.517, 95% CI: 0.317–0.841, *p* = 0.008) and focal epilepsy (*B* = −0.478, OR = 0.620, 95% CI: 0.403–0.954, *p* = 0.030). Forgetfulness (*n* = 91, 32.5%).
Kumar et al. (2021)	India	Cross‐sectional study	Adults (*n* = 268)	157 men	4‐point Morisky	30% nonadherent	Polytherapy, adverse events, treatment duration ≥ 3 years	Treatment/disease	AED polytherapy (OR = 4.5, 95% CI: 2.1–9.5, *p* = 0.001), drug‐related adverse events (OR = 3.9, 95% CI: 2.1–7.3, *p* = 0.001), and treatment duration > 3 years (OR = 2.6, 95% CI: 1.3–5.0, *p* = 0.003).
Korkmaz et al. (2020)	Türkiye	Cross‐sectional study	Caregivers of children (*n* = 226)	116 men	MMAS‐8; PHLKS (Public Health Literacy Knowledge Scale)	47.3% high adherence	Caregiver health literacy, forgetfulness, child's age	Patient/social and economic	Drug‐resistant epilepsy (*B* = −0.29, OR = 0.8, 95% CI: 0.3–2.0, *p* = 0.6) and Public Health Literacy Knowledge Scale (*B* = −0.2, OR = 0.8, 95% CI: 0.7–0.9, *p* = 0.008). 6–11 years (middle childhood) (*B* = 0.56, OR = 1.7, 95% CI: 0.9–3.5, *p* = 0.1), 12–18 years (adolescent) (*B* = 1.08, OR = 2.9, 95% CI: 1.4–6.5, *p* = 0.007), caregiver's educational status (*B* = 0.25, OR = 1.3, 95% CI: 0.7–2.5, *p* = 0.5), and duration of epilepsy (*B* = 0.23, OR = 1.3, 95% CI: 0.7–2.4, *p* = 0.5). Forgetfulness (33.6%).
Dayapoğlu et al. (2021)	Türkiye	Descriptive, cross‐sectional	Adults (*n* = 174)	91 men	MMAS‐8; BMQ‐T (Beliefs about Medicines Questionnaire)	Not specified	Perception of medication overuse and harm	Patient‐related	General overuse (*B* = −0.923, *β* = −0.331, *t* = −4.997, *p* < 0.001) and general harm (*B* = −0.781, *β* = −0.517, *t* = −8.607, *p* < 0.001).
Narducci et al. (2023)	Italy	Cross‐sectional study	Adults (*n* = 200)	72 male	Morisky Medication Adherence Scale (MMAS‐8), Quality of Life in Epilepsy Inventory 31 (QoLIE‐31), Beck Depression Inventory‐II (BDI‐II), Generalized Anxiety Disorder‐7 (GAD‐7), and 14‐item Resilience Scale (RES14)	48 (24%) PwE had low adherence (MMAS‐8 < 6), 143 (71.5%) had medium adherence (MMAS‐8 score between > 6 and < 8), 9 (4.5%) PwE showed high adherence (MMAS‐8 ≥ 8).	Female gender, age, psychological flexibility, depression, anxiety	Patient‐related	Women were more adherent to medication (*p*‐value = 0.035). Morisky Medication Adherence Scale‐8 showed a direct correlation with RES14 (*p*‐value = 0.001) and age (*p*‐value = 0.001), while inversely correlated with BDI‐II (*p*‐value = 0.001) and GAD‐7 (*p*‐value = 0.001).
Guo et al. (2015)	China	Cross‐sectional study	Adults (*n* = 184)	100 male	MMAS‐8; the Beck Depression Inventory (BDI) and the Beck Anxiety Inventory (BAI).	The MMAS‐8 indicated that 39.7% of the patients had low adherence, 34.2% had moderate adherence, and 26.1% had high adherence.	Moderate‐to‐severe depression and anxiety	Patient‐related	Moderate‐to‐severe depression (*r* = −0.281, *χ*² = 13.623, *p* < 0.001) and moderate‐to‐severe anxiety (−0.255, *χ*² = 8.331, *p* = 0.004).
Verma et al. (2018)	India (rural)	Cross‐sectional study	Adults (*n* = 230)	139 male	Morisky–Green; BMQ (Beliefs about Medicine Questionnaire)	57% nonadherent, 49.6% had moderate and 7.4% had low adherence levels.	Low SES, seizure severity, duration, relapse, adverse effects	Social and economic/disease/treatment	Socioeconomic Class V (OR: 23, 95% CI: 7.2–77.1; *p* < 0.001) and one or more seizure in the past 6 months (OR: 32.25, 95% CI: 14.57–71.42; *p* < 0.001). Potential adverse effects of AEDs (*p* < 0.0013).
Abebe et al. (2022)	Ethiopia	Cross‐sectional study	Adults (*n* = 187)	98 male	MMAS‐8	Not specified	Monthly income, psychiatric illness perception, seizure‐free < 1 year	Social and economic/patient/disease	Monthly income > 3000 ETB (AOR = 0.164, 95% CI: 0.038–0.702), monthly income 2000–3000 ETB (AOR = 0.110, 95% CI: 0.026–0.461), perceived epilepsy as a psychiatric disorder (AOR = 0.250, 95% CI: 0.087–0.716), and seizure‐free period < 1 year (AOR = 0.206, 95% CI: 0.076–0.562).
Dabilgou et al. (2025)	Burkina Faso	Cross‐sectional study	Adults (*n* = 107)	51 male	MMAS	73.8% nonadherent	Rural residence, divorce, low education, stigma, financial insufficiency, forgetfulness	Social and economic/patient	Urban residence (OR = 0.105, 95% CI: 0.015–0.729, *p* = 0.023), marital status—married (OR = 0.001, 95% CI: 0–0.091, *p* = 0.002), education level—primary (OR = 0.085, 95% CI: 0.009–0.77, *p* = 0.028), stigmatization (OR = 2.32, 95% CI: 1.105–4.87, *p* = 0.026), and duration of epilepsy < 5 years (OR = 0.006, 95% CI: 0–0.439, *p* = 0.019). Divorce (*p* = 0.002). 73, forgetfulness in 71 (89.9%).
Yang et al. (2023)	China	Cross‐sectional study	Children (*n* = 1847)	983 male	MMAS	38% poor adherence	Younger age, city residence, new diagnosis, access barriers	Patient/social and economic/health system	Age (odds ratio [OR] = 1.117, 95% CI: 1.084–1.151), being an only child (OR = 0.749, 95% CI: 0.604–0.927), place of residence (OR = 0.703, 95% CI: 0.535–0.923), annual medical expense (OR = 0.615 95% CI: 0.387–0.977), being newly diagnosed (OR = 1.685, 95% CI: 1.344–2.113), and comorbidities (OR = 0.783, 95% CI: 0.629–0.974).
Choi et al. (2023)	ABD, New York	Cross‐sectional study	Adults (*n* = 644)	239 male	MMAS‐8; BIPQ (Brief Illness Perception Questionnaire)	23% high adherence	Understanding of epilepsy, emotional impact, stigma, depression	Patient/social and economic	Understanding of epilepsy (OR = 1.17, 95% CI: 1.07–1.27, *p* < 0.001).
Dima and Shibeshi (2022)	Ethiopia	Cross‐sectional study	Children (*n* = 192)	128 male	MMAS	65% adherent	Family size ≤ 5, short duration, low income, recent seizures	Social and economic/disease	Family size ≤ 5 (+), duration < 1 year (+), low income (−), recent seizure (−), overall effect (OR = 0.89, 95% CI: 0.82–0.97, *p* = 0.01), and emotion (OR = 0.94, 95% CI: 0.86–0.99, *p* = 0.03).
Afsharkhas et al. (2022)	Iran	Cross‐sectional study	Children (*n* = 120)	75 male	Modified Morisky	41.7% high adherence	Parent education, perinatal morbidity, travel, forgetfulness (30%)	Patient/social and economic/disease	More educated fathers (*p* = 0.002), more educated mothers (*p* = 0.035), absence of perinatal morbidities (*p* = 0.001), and type of seizure (*p* = 0.022; focal impaired awareness seizures 57.1%, atonic seizures 11.1%).
Özer et al. (2023)	Türkiye	Cross‐sectional study	Adults (*n* = 150)	71 male	MMAS‐8; Fatalism Scale (FS), Holistic Complementary and Alternative Medicine Questionnaire (HCAMQ)	Not specified	Fatalism, pessimism, belief in predetermination	Patient‐related	Primary education graduate (*β* = −0.234, *p* < 0.01) and FS/fatalism scale (*β* = −0.509, *p* < 0.01). Mean scores of the participants: 3.26 ± 0.71 for predetermination, 3.33 ± 0.97 for luck, and 3.59 ± 0.89 for pessimism.
Fadare et al. (2018)	Nigeria	Cross‐sectional study	Children (*n* = 126)	59 male	MMAS; LAEP (Liverpool Adverse Effect Profile)	44.5% low adherence, 17.2 high, 38.3 medium adherence	Side effects (fatigue, headache)	Treatment‐related	The most common reported adverse effects among respondents were tiredness (30.4%) and headache (22.5%) (−).
Chauhan et al. (2018)	India	Cross‐sectional study	Children/caregivers (*n* = 112)	62 male	MMAS‐8 Treatment Satisfaction Questionnaire (TSQM 1.4	Fifty (44.6%) patients had low adherence, 29 (25.9%) medium adherence, and 33 (29.5%) high adherence to AEDs as per MMAS‐8 questionnaire. Altogether 79 (70.5%) children were nonadherent when dichotomized MMAS scores were considered.	Child age, duration, family size, frequency of intake, satisfaction	Patient/social and economic/treatment	Age (*p* = 0.0003), duration of epilepsy (*p* = 0.0075), family history of epilepsy (*p* = 0.0370), family size (*p* = 0.0401), mean number of family members (*p* = 0.0118), medication timing frequency (*p* = 0.0228), and employment status of caregiver (*p* = 0.0452). Global satisfaction (*p* = 0.0152).
Tan et al. (2015)	Malaysia	Cross‐sectional study	Adults (*n* = 145)	74 male	MMAS‐8	64.1% poor adherence	Younger age, side effects, short duration, necessity uncertainty	Patient/treatment/disease	Younger age (*χ*² = 7.609, *p* = 0.022), medication adverse effects (*χ*² = 5.075, *p* = 0.020), shorter duration of epilepsy (*r* = 0.180, *p* = 0.030), and uncertainty about the necessity for AEDs (*χ*² = 11.803, *p* = 0.001).
Mamo et al. (2024)	Ethiopia	Cross‐sectional study	Adults (*n* = 214)	99 male	MMAS‐4; MOCA‐B (Montreal Cognitive Assessment tool)	54.2% poor adherence	Educational level, cognitive impairment, physical exercise	Patient/social and economic	Education (AOR = 0.04, 95% CI: 0.02–0.14, *p* = 0.001), primary education (AOR = 0.32, 95% CI: 0.15–0.70, *p* = 0.004), physical exercise—sometimes (AOR = 16.30, 95% CI: 1.24–214.99, *p* = 0.034), and MMAS‐4 score = 0 (AOR = 12.0, 95% CI: 1.53–93.75, *p* = 0.018). Lower cognitive function levels (AOR = 12.0, 95% CI (1.53, 93.75), *p* = 0.018).
Yang et al. (2020)	China	Cross‐sectional study	Children (*n* = 399)	230 male	MMAS‐8	21.3% (85/399) patients showed good adherence, 51.4% (205/399) moderate adherence, and 27.3% (109/399) poor adherence.	Patient age, household income, source of drug information	Patient/social and economic/health system	Age (*B* = −0.079, OR = 0.92, 95% CI: 0.88–0.97, *p* = 0.001), symptomatic epilepsy (*B* = −0.636, OR = 0.53, 95% CI: 0.35–0.80, *p* = 0.002), total household income 5000–10,000 RMB (*B* = 1.007, OR = 2.74, 95% CI: 1.54–4.88, *p* = 0.001), and hospital as source of drug information (*B* = 1.272, OR = 3.57, 95% CI: 1.08–11.76, *p* = 0.036).
Yang et al. (2025)	Cross‐sectional study	Cross‐sectional study	Children (*n* = 1357)	718 male	MMAS	Adherence was 73.4% (996/1357)	Only child status, caregiver unemployment	Patient/social and economic	Younger patient age (*β* = −0.064; standard error [SE] = 0.008; *p* = 0.016), being an only child (*β* = −0.071; SE = 0.062; *p* = 0.008), caregiver unemployment (*β *= −0.091; SE = 0063; *p* = 0.001), and comorbidities (*β* = 0.186; SE = 0.061; *p* = 0.000).
Hasiso and Desse (2016)	Ethiopia A25:B25	Cross‐sectional study	Adults (*n* = 194)	109 male	MMAS‐8	32% adherent	Forgetfulness, running out of pills, duration, education, comorbidity, married status	Patient/social and economic/health system	Forgetfulness 49 (75.4%), run out of pills 7 (10.8%), duration of epilepsy treatment < 1 year (*p* = 0.011), 1–3 years (*p* = 0.002), 3–5 years (*p* = 0.007), married status (*p* = 0.006), educational status grade 9–12 (*p* = 0.028), college/university education (*p* = 0.002), and no comorbidity (*p* = 0.008).
Akoluk and Ovayolu (2021)	Türkiye	Cross‐sectional study	Adults (*n* = 325)	189 male	Morisky Scale	27.7% of the patients were high, 58.5% were moderate, and 13.8% were low.	Age, gender, education, income, number of drugs	Patient/social and economic/treatment	Age (*χ*² = 26.440, *p* = 0.001), gender (*χ*² = 13.069, *p* = 0.001), educational status (*χ*² = 30.636, *p* < 0.001), occupation (*χ*² = 25.314, *p* = 0.005), place of residence (*χ*² = 31.824, *p* < 0.001), economic status (*χ*² = 22.828, *p* < 0.001), number of medications used (*χ*² = 23.715, *p* = 0.001), and action taken when experiencing medication‐related problems (*χ*² = 15.692, *p* = 0.003).
Asghar et al. (2021)	Pakistan	Cohort study	Cohort (*n* = 11,490)	5883 male	Records	Variable	Age, comorbidities, drug type	Patient/treatment	Age (OR = 2.04; 95% CI: 1.61–2.58), migraine (OR = 2.21; 95% CI: 1.79–2.74), psychiatric disorders (OR = 4.28; 95% CI: 3.63–4.93), other comorbidities (OR = 1.52; 95% CI: 1.29–1.75), levetiracetam (OR = 0.69; 95% CI: 0.55–0.86), valproic acid (OR = 0.52; 95% CI: 0.46–0.58), carbamazepine (OR = 0.81; 95% CI: 0.69–0.96), lamotrigine (OR = 0.80; 95% CI: 0.68–0.95), lacosamide (OR = 0.65; 95% CI: 0.51–0.82), and other antiepileptic drugs (OR = 2.35; 95% CI: 1.93–2.88) were identified as significant variables.
Teh et al. (2020)	Malaysia	Cross‐sectional study	Adults (*n* = 208)	87 male	MCQ (Medication Compliance Questionnaire); B‐IPQ (Brief Illness Perception Questionnaire)	Adherent: 119 (57.2%), nonadherent: 89 (42.8%)	Employment/student status, pharmacy access	Patient/social and economic/health system	Significant variables associated with nonadherence included employed/student status (AOR = 2.26; 95% CI: 1.19–4.29; *p* = 0.012) and average or below‐average perceived access to pharmacy services (Ahttps://www.who.int/news‐room/fact‐sheets/detail/epilepsyAOR = 2.94; 95% CI: 1.38–6.24; *p* = 0.005).
Sunny et al. (2020)	India	Cross‐sectional study	Adults (*n* = 304)	185 male	MMAS‐8; BMQ (Beliefs about Medicines Questionnaire)	Adherent group (*n* = 135, 44.4%) and a nonadherent group (*n* = 169, 55.6%).	Income, side effects, seizure frequency, ASM (antiseizure medication) cost/access	Social and economic/treatment/health system	Income (*β* = −0.215, *p* < 0.001), ASM side effects (*β* = 0.177, *p* = 0.001), seizure frequency (*β* = 0.167, *p* = 0.002), ASM availability (*β* = −0.151, *p* = 0.004), cost of ASMs (*β* = −0.134, *p* = 0.013), and NCD (necessity‐concern differential) (*β* = −0.184, *p* = 0.001) were identified as significant predictors.
Solomon et al. (2023)	Ethiopia	Cross‐sectional study	Adults (*n* = 352)	216 male	MMAS‐8; Oslo‐3	40% nonadherent	Social support, stigma, joblessness, polytherapy, divorce	Social and economic/treatment	Significant factors included female sex (AOR = 3.37, 95% CI: 1.84–6.18), divorced status (AOR = 9.13, 95% CI: 1.80–46.34), unemployment (AOR = 7.33, 95% CI: 3.24–16.56), perceived poor social support (AOR = 2.73, 95% CI: 1.28–5.82), perceived stigma (AOR = 5.07, 95% CI: 2.40–10.68), polytherapy (AOR = 2.23, 95% CI: 1.06–4.71), drug side effects (AOR = 6.03, 95% CI: 3.17–11.45), buying medications (AOR = 5.81, 95% CI: 3.63–16.79), and duration of treatment (AOR = 4.31, 95% CI: 1.863–9.97).
Motioleslam et al. (2024)	Iran	Cross‐sectional study	Adults (*n* = 250)	119 male	Morisky; Rosenberg Self‐Esteem Scale	Low adherence: 116 participants (46.4%), medium adherence: 76 participants (30.4%), and high adherence: 58 participants (23.2%), among a total of 250 participants.	Self‐esteem, gender, educational level	Patient‐related	Significant correlates of medication adherence included self‐esteem (*r* = 0.464, *p* < 0.001), gender (*r* = 0.011, *p* < 0.001), marital status (*r* = −0.104, *p* < 0.001), educational level (*r* = 0.413, *p* < 0.001), and duration of treatment (*r* = 0.341, *p* < 0.001). Employment status was not statistically significant (*r* = −0.053, *p* = 0.404).
Khatooni et al. (2024)	Iran	Cross‐sectional study	Adults (*n* = 235)	139 male	MMAS‐8; DASS‐21	61/8% of the participant had proper medication adherence and 38/3% had poor medication adherence.	Severe depression and anxiety	Patient‐related	Severe depression (AOR = 3.45, 95% CI: 1.16–4.01, *p* = 0.045), extremely severe depression (AOR = 3.83, 95% CI: 1.91–7.25, *p* < 0.001), severe anxiety (AOR = 2.08, 95% CI: 1.71–6.05, *p* = 0.027), and extremely severe anxiety (AOR = 2.88, 95% CI: 1.81–6.45, *p* = 0.001).
Verma et al. (2020)	India	Cross‐sectional study	Adults (*n* = 385)	252 male	MMAS‐8; BMQ (Beliefs about Medicines Questionnaire)	58.8% nonadherent	Side effect concerns, socioeconomic status, necessity doubts, seizure frequency	Social and economic/treatment/patient	Significant associations were identified for necessity beliefs with age (*r* = −0.628, *p* < 0.0001) and seizure frequency (*r* = −0.156, *p* = 0.02). Significant associations were also found for concern beliefs with socioeconomic status (*r* = 0.200, *p* < 0.001) and seizure frequency (*r* = 0.397, *p* < 0.0001).
Chen et al. (2010)	Taiwan	Cross‐sectional study	Adults (*n* = 357)	193 male	AEP (the Adverse Events Profile); ESES (Epilepsy Self‐Efficacy Scale), rust in Physician Scale	Not specified	Gender, comorbidity, self‐efficacy, dosing frequency	Patient/disease/treatment	Gender (male) (*p* = 0.001), comorbid chronic diseases (OR = 2.11, 95% CI: 1.07–4.15, *p* = 0.03), self‐driving (OR = 0.33, 95% CI: 0.18–0.62, *p* < 0.01), seizure after a missed dose (OR = 0.20, 95% CI: 0.09–0.44, *p* < 0.01), and self‐efficacy (OR = 2.98, 95% CI: 1.71–5.21, *p* < 0.01). Daily drug dosing frequency (*p* = 0.003).
Büyükbayram et al. (2023)	Türkiye	Cross‐sectional study	Adults (*n* = 220)	118 men	MMAS‐8; CES (Concealment of Epilepsy Scale)	72.3% low adherence	Gender, employment, seizure count	Patient/social and economic/disease	Significant factors affecting medication adherence included CES (*β* = −0.351, *p* < 0.001), female gender (*β* = 0.136, *p* = 0.016), employment status (employed) (*β* = 0.295, *p* < 0.001), monotherapy (*β* = 0.174, *p* = 0.004), number of seizures in the last year (*β* = −0.133, *p* = 0.036), and disease duration of 11 years or longer (*β* = 0.165, *p* = 0.007).
Almwled et al. (2022)	Saudi Arabia	Cross‐sectional study	Adults (*n* = 207)	96 male	VAS (Visual Analog Scale); PHQ‐9 (Patient Health Questionnaire‐9); MOCA (Montreal Cognitive Assessment)	81.6% mean adherence	Depression, anxiety, physical symptoms, educational level, type of medication	Patient‐related	Significant variables associated with treatment adherence were PHQ9 score (*β* = −0.052, *p* = 0.011), GAD7 score (*β* = −0.064, *p* = 0.005), and PHQ15 score (*β* = −0.071, *p* = 0.001). In multivariate analysis, only PHQ15 score remained significant (estimate = −0.218, *p* = 0.020), educational level (*p* = 0.003), lamotrigine (*p* = 0.004).
Chesaniuk et al. (2014)	Colombia	Cross‐sectional study	Adults (*n* = 140)	53 male	Epilepsy Stigma Scale, Knobel brief adherence questionnaire	Not specified	Stigma (mediated by knowledge, motivation, skills)	Social and economic/patient	Perceived epilepsy‐related stigma (*r* = −0.18, *p* < 0.05), information (*r* = −0.28, *p* < 0.05), motivation (*r* = −0.55, *p* < 0.05), and behavioral skills (*r* = −0.41, *p* < 0.05) were significantly associated with medication adherence.
Liu (2013)	China	Cross‐sectional study	All ages (*n* = 368)	217 male	Own survey	177 (48.1%) patients were categorized as nonadherent and 191 (51.9%) as adherent.	Duration of illness, forgetfulness, attitude	Disease/patient/social and economic	A significant difference was obtained between adherence and duration of illness (*p* = 0.007, *t* = −2.738). Of the patients who did not adhere to drugs (69.6%), the primary reason was forgetfulness (65.8%) or not having medication on hand (3.8%), followed by patients’ negative attitude toward the therapy (12.8%), a bad patient–prescriber relationship (9.5%), side effects or worry about side effects (5.4%), inability to buy drugs (1.9%), and other reasons such as taste of medication or requirement for drug storage (0.8%).
Getnet et al. (2016)	Ethiopia	Cross‐sectional study	Adults (*n* = 450)	264 male	MMAS‐8	37.8% nonadherence	Treatment duration ≥ 6 years, payment difficulty, knowledge, stigma	Disease/social and economic/patient	Being on treatment for 6 years and above (AOR = 3.47, 95% CI: 1.88–6.40), payment for AEDs (AOR = 2.76, 95% CI: 1.73–4.42), lack of health information (AOR = 2.20, 95% CI: 1.41–3.43), poor social support (AOR = 1.88, 95%, CI: 1.01–3.50), perceived stigma (AOR = 2.27, 95% CI: 1.45–3.56), and experienced side effect (AOR = 1.70, 95% CI: 1.06–2.72) were significantly associated with antiepileptic drug nonadherence.
Niriayo et al. (2019)	Ethiopia	Cross‐sectional study	Adults (*n* = 292)	179 male	The Belief about Medicines Questionnaire (BMQ), self‐reported medication adherence questionnaire	65.4% nonadherent	Comorbidity, recent seizures, negative drug beliefs, forgetfulness	Disease/patient	Comorbidity (AOR: 3.51, 95% CI: 1.20–10.31), seizure encounter within the last 3 months (AOR: 5.45, 95% CI: 2.48–12.00), low medication necessity belief (AOR: 3.38, 95% CI: 1.14–10.00), high medication concern belief (AOR: 4.23, 95% CI: 2.07–8.63), and negative medication belief (AOR: 4.17, 95% CI: 1.74–10.02) were predictors of medication nonadherence.
Lin et al. (2018)	Iran	Cross‐sectional study	Adults (*n* = 760)	346 male	Duke University Religion Index; DUREL, Brief Religious Coping Scale; BriefRCOPE, MARS‐5, Quality of Life in Epilepsy; QOLIE‐31	Not specified	Religious coping methods, religiosity	Patient‐related	Negative religious coping was associated with medication adherence (standardized coefficient = −0.060, *p* < 0.001). Medication adherence was also associated with quality of life (standardized coefficient = 0.052, *p* = 0.01), and negative religious coping together with medication adherence was associated with quality of life (standardized coefficient = 0.015, *p* = 0.02).
Memiş and Koç (2020)	Türkiye	Cross‐sectional study	Children/parents (*n* = 125)	77 male	Epilepsy Knowledge Test for Parents	Not specified	Parental education, lack of knowledge, forgetfulness	Patient/social and economic	Mother's education level and use of non‐pharmacological methods showed a significant association (*p* = 0.001). Father's education level and use of non‐pharmacological methods also showed a significant association (*p* = 0.002). The difference in epilepsy knowledge scores between parents who discontinued medication after seizures ended and those who did not was not statistically significant (*p* = 0.055). Forgetfulness (28.8%, *n* = 15).
Suzuki et al. (2020)	Japan	Cross‐sectional study	Adults (*n* = 855)	442 male	Morisky Medication Adherence Scale	Not specified	Forgetfulness, dementia, young age, polytherapy (≥ 3), living alone	Patient/disease/treatment	Gender (female) (OR = 1.42, 95% CI: 1.07–1.89, *p* = 0.014), dementia (OR = 2.69, 95% CI: 1.52–4.76, *p* = 0.001), use of two ASMs (OR = 1.52, 95% CI: 1.09–2.12, *p* = 0.014), younger age (*p* < 0.05), and living alone (OR = 2.09, 95% CI: 0.39–3.16, *p* = 0.001).
Taha et al. (2024)	Scotland	Cohort	Adults (*n* = 266)	168 male	Electronic records	20.3% poor adherence	Mental illness, substance use, lack of medication	Patient/health system	Medication possession ratio below 80%: 54/266 (20.3%), poor medication adherence among patients with a history of mental illness: 22/93 (24%), poor medication adherence among patients with a history of alcohol and/or recreational drug use: 22/66 (33%), patients with a rescue medication protocol: 39/219 (17.8%).

#### 3.3.1 Patient‐Related Factors

Across all populations, forgetfulness is identified as the primary cause of nonadherence, with rates ranging approximately from 20% to 90% (Dima and Shibeshi 2022; Gurumurthy et al. 2017; Korkmaz et al. 2020; Suzuki et al. 2020; Dabilgou et al. 2025; Liu et al. 2013). Age has been found to influence medication adherence; adherence tends to increase in older adults (Akoluk and Ovayolu 2021; Narducci et al. 2023; Choi et al. 2023), while nonadherence is more common among younger individuals (Ferrari et al. 2013; Suzuki et al. 2020; Tan et al. 2015). Gender, marital status (married, divorced, single), and low educational level are associated with increased nonadherence (Solomon et al. 2023; Dabilgou et al. 2025; Mamo et al. 2024), with some studies reporting better adherence among females (Narducci et al. 2023; Büyükbayram et al. 2023). However, certain studies have reported no significant effect of sociodemographic variables such as gender and age on nonadherence (Liu et al. 2013).

Significant associations have been observed between depression and anxiety levels and poor medication adherence (Getnet et al. 2016; Guo et al. 2015; Khatooni et al. 2024). Depression, anxiety, and physical symptoms exacerbate nonadherence (Almwled et al. 2022). Conversely, high levels of self‐esteem have been linked to improved adherence (Motioleslam et al. 2024). In pediatric populations, increased self‐efficacy positively influences adherence (Yang et al. 2025). Medication adherence is negatively correlated with beliefs related to medication “overuse/harm”; specifically, low necessity beliefs, high medication concerns, and negative medication beliefs increase nonadherence (Dayapoğlu et al. 2021; Niriayo et al. 2019). Positive illness perceptions—particularly regarding the overall impact of epilepsy on life, understanding of epilepsy, and its emotional effects—are associated with higher adherence levels (Choi et al. 2023). Additionally, motivation and behavioral skills have been found to enhance treatment adherence (Chesaniuk et al. 2014). Patients who perceive epilepsy as a psychiatric disorder tend to be more nonadherent compared to those who perceive it as a neurological condition (Abebe et al. 2022). In pediatric samples, a positive correlation exists between the number of family members and treatment nonadherence (Chauhan et al. 2018). Furthermore, inability to administer treatment to the patient (23.0%), parental forgetfulness in giving medication (18.3%), and failure to take medication while traveling or leaving home have been identified as factors contributing to nonadherence (Afsharkhas et al. 2022). In pediatric populations, age and caregivers’ health literacy are negatively correlated with nonadherence (Korkmaz et al. 2020).

#### 3.3.2 Treatment‐Related Factors

The complexity of the medication regimen and adverse effects negatively impacted treatment adherence (Ferrari et al. 2013; Solomon et al. 2023). Reported side effects included sedation, fatigue, headache, dizziness, and cognitive slowing (Solomon et al. 2023; Getnet et al. 2016; Verma et al. 2018; Tan et al. 2015; Fadare et al. 2018; Kumar et al. 2021). A higher number of medications and lack of information about the drugs increased nonadherence (Akoluk and Ovayolu 2021; Solomon et al. 2023). Treatment duration exceeding 3 years was associated with nonadherence (Kumar et al. 2021). In pediatric samples, a negative correlation was found between medication frequency and adherence (Chauhan et al. 2018).

#### 3.3.3 Disease‐Related Factors

The clinical course of epilepsy significantly influences medication adherence. Higher seizure frequency, longer epilepsy duration, and greater seizure severity were consistently associated with lower adherence levels (Dima and Shibeshi 2022; Verma et al. 2018; Choi et al. 2023; Sunny et al. 2020). Patients who experienced recent seizures or poor seizure control often demonstrated reduced motivation to follow treatment regimens, whereas those with longer seizure‐free periods tended to show better adherence (Dima and Shibeshi 2022; Choi et al. 2023; Niriayo et al. 2019). Individuals who did not experience seizures after missed doses were more likely to maintain adherence, suggesting that awareness of the consequences of nonadherence may reinforce medication‐taking behaviors (Chen et al. 2010). Although seizure type did not demonstrate a significant relationship with adherence (Liu et al. 2013), the overall disease burden—including seizure frequency, severity, and duration—remains a key determinant of consistent medication use (Verma et al. 2018; Büyükbayram et al. 2023).

#### 3.3.4 Social and Economic Factors

Low socioeconomic status, treatment costs, and living in rural areas negatively affected treatment adherence (Gurumurthy et al. 2017; Dabilgou et al. 2025; Choi et al. 2023; Verma et al. 2020; Yang et al. 2020; Yang et al. 2023; Asghar et al. 2021). No significant difference in adherence was found between urban and rural residences (Liu et al. 2013). Individuals who were employed or students exhibited lower adherence levels compared to those who were unemployed, retired, or homemakers (Getnet et al. 2016). A relationship was identified between poor social support, perceived stigma, and low adherence (Chesaniuk et al. 2014). Within cultural factors, a fatalistic attitude was associated with reduced treatment adherence (Özer et al. 2023). Conversely, positive religious coping strategies were linked to higher adherence (Lin et al. 2018). In pediatric samples, family communication, problem‐solving skills, and parental knowledge levels were found to be related to adherence (Dima and Shibeshi 2022). Smaller family size (fewer than five members) and shorter diagnosis duration (less than 1 year) were associated with higher adherence (Dima and Shibeshi 2022). The high value placed on children within Turkish family structures was also linked to better adherence (Memiş and Koç 2020). A low income level was correlated with nonadherence (Akoluk and Ovayolu 2021). In pediatric samples, caregivers’ unemployment was positively associated with adherence (Yang et al. 2025).

#### 3.3.5 Health System and Healthcare Provider‐Related Factors

Access to healthcare services and the performance of the health system were found to significantly impact treatment adherence. In a pediatric sample, difficulties faced by parents in making appointments and accessing healthcare services were reported to be associated with nonadherence to treatment (Yang et al. 2023). Individuals with medication possession rates below 80% experienced worse clinical outcomes, which may be linked to the lack of systematic follow‐up (Taha et al. 2024). Another study identified that perceived access to pharmacy services at average or below‐average levels was associated with poor adherence. Participants reported issues such as long waiting times and overcrowding in outpatient pharmacy departments, lack of parking, and long distances to the hospital (Teh et al. 2020). A study conducted in India found that medication costs increased the likelihood of treatment nonadherence (Sunny et al. 2020).

## 4 Discussion

This systematic review comprehensively evaluated the factors associated with medication adherence among epilepsy patients and showed that adherence may be influenced by a multidimensional interplay of patient‐related, treatment‐related, disease‐related, social, and health system factors. Although adherence levels varied across countries, studies consistently indicated moderate adherence influenced not by any single determinant but by the interaction between psychological states, socioeconomic challenges, treatment burden, cultural beliefs, and health system support (Gurumurthy et al. 2017; Ferrari et al. 2013; Dayapoğlu et al. 2021). Following the WHO adherence model (Sabaté 2003; Peh et al. 2021), this discussion integrates findings across these domains to illustrate how adherence behaviors emerge from complex contextual dynamics.

### 4.1 Patient‐Related Factors

Patient‐related determinants—including behavioral patterns, psychological well‐being, demographic characteristics, and illness beliefs—were frequently associated with medication adherence across studies. Forgetfulness emerged as a common behavioral barrier, reported in 20%–90% of cases (Dima and Shibeshi 2022; Gurumurthy et al. 2017; Korkmaz et al. 2020; Dabilgou et al. 2025), reflecting cognitive load, daily routines, and treatment fatigue rather than simple patient negligence. Sociodemographic findings were inconsistent: While some studies identified higher adherence among women, married individuals, and older adults (Ferrari et al. 2013; Solomon et al. 2023; Büyükbayram et al. 2023), other studies reported no significant associations with demographic characteristics (Liu et al. 2013), suggesting that demographics may exert context‐dependent rather than universal effects. Psychological factors, including depression and anxiety, showed strong negative associations with adherence (Getnet et al. 2016; Khatooni et al. 2024), whereas higher self‐efficacy and self‐esteem were associated with better adherence (Motioleslam et al. 2024; Yang et al. 2025). Illness perceptions were also influential; patients who viewed epilepsy as a neurological condition were more adherent than those with psychiatric or spiritual interpretations (Choi et al. 2023; Abebe et al. 2022). Among pediatric patients, caregiver‐related issues—such as forgetfulness, household size, or disruptions during travel—were significantly associated with poor adherence (Chauhan et al. 2018; Afsharkhas et al. 2022), highlighting the importance of family‐centered interventions.

#### 4.1.1 Treatment‐Related Factors

Treatment‐related influences—such as medication regimen complexity, side effects, and treatment duration—were consistently associated with adherence. Polytherapy, higher dosing frequency, and long‐term treatment were frequently associated with nonadherence (Akoluk and Ovayolu 2021; Ferrari et al. 2013; Solomon et al. 2023), mirroring patterns seen in other chronic diseases where complex regimens may impose additional cognitive and logistical burdens. Adverse drug effects, including sedation, dizziness, fatigue, and cognitive slowing, were associated with disruptions in daily functioning and lower motivation for treatment continuation (Getnet et al. 2016; Tan et al. 2015), especially among individuals with work responsibilities requiring alertness. Poor adherence was closely associated with suboptimal seizure control and increased generalized seizure risk (Suzuki et al. 2020), and prolonged treatment duration (> 3 years) was associated with medication fatigue and reduced perceived necessity of consistent drug use (Kumar et al. 2021). These findings highlight the importance of personalized treatment planning and proactive side‐effect management.

#### 4.1.2 Disease‐Related Factors

Disease‐related characteristics—including seizure frequency, severity, and recent seizure history—were consistently associated with adherence patterns. Patients with recent seizures exhibited lower adherence (Dima and Shibeshi 2022; Niriayo et al. 2019), which may be associated with increased psychological distress, reduced confidence in treatment effectiveness, or discouragement following breakthrough seizures. Conversely, seizure freedom was associated with higher adherence (Choi et al. 2023), suggesting a possible relationship between perceived treatment effectiveness and sustained medication use. High seizure frequency and severity were also associated with nonadherence (Verma et al. 2018; Sunny et al. 2020), potentially related to fatigue, functional limitations, and heightened fear. However, some patients with mild seizure profiles still demonstrated poor adherence (Chen et al. 2010), indicating that perceived severity may differ from clinical severity. Annual seizure frequency demonstrated consistent associations with adherence (Büyükbayram et al. 2023), whereas seizure type did not (Liu et al. 2013), emphasizing that overall disease experience may be more relevant to adherence behaviors than seizure classification alone.

### 4.2 Social and Economic Factors

Social and economic determinants varied widely across studies, reflecting different cultural and health system contexts. Low socioeconomic status, high treatment costs, and financial strain were associated with poor adherence (Gurumurthy et al. 2017; Yang et al. 2023; Asghar et al. 2021), although in some regions these factors were less influential due to subsidized medication programs or strong family support systems. An interesting finding was that caregiver unemployment was associated with better adherence in pediatric cases (Yang et al. 2025), possibly because available caregiver time compensated for financial limitations. Social stigma, insufficient support, and cultural beliefs were negatively associated with adherence (Chesaniuk et al. 2014; Özer et al. 2023), whereas positive religious coping was associated with better adherence outcomes (Lin et al. 2018). Family functioning—including communication quality, problem solving, and health literacy—was especially important for pediatric adherence (Dima and Shibeshi 2022; Memiş and Koç 2020), and the strong cultural emphasis on child welfare in countries such as Türkiye may be associated with higher adherence rates reported in local studies.

### 4.3 Health System and Healthcare Provider‐Related Factors

Health system issues—including accessibility, continuity of care, and service organization—were prominent factors associated with adherence outcomes. Difficulties with appointments, transportation challenges, and inconsistent medication supply were frequently associated with adherence issues among caregivers of pediatric patients (Yang et al. 2023), indicating that adherence should be understood not only as an individual behavior but also as a system‐level outcome. Low medication possession rates (< 80%) were associated with worse clinical outcomes (Taha et al. 2024), underscoring the importance of reliable pharmaceutical supply and timely follow‐up. Negative pharmacy experiences—such as long waiting times and limited accessibility—were associated with decreased adherence (Teh et al. 2020), and in low‐ and middle‐income settings high drug costs were associated with treatment interruptions (Sunny et al. 2020). These findings highlight the need for health systems to improve logistical efficiency, service quality, and affordability to support sustained medication adherence.

### 4.4 Heterogeneity of Evidence Across Subgroups

Considerable heterogeneity was observed across the included studies in terms of population characteristics, geographic and economic settings, study design, and measurement tools. When interpreted in subgroup contexts, distinct patterns emerged. Pediatric studies, particularly those involving caregivers, more frequently highlighted the role of family dynamics, caregiver burden, and household‐related factors, whereas adult populations showed stronger associations with psychological distress, treatment complexity, and individual beliefs about illness. In addition, socioeconomic and health system‐related barriers were more prominent in low‐ and middle‐income countries, particularly regarding medication cost and access to care, while these factors were less dominant in high‐income settings. Differences in adherence measurement tools (e.g., MMAS‐4 vs. MMAS‐8) contributed to variability in reported adherence levels, although the direction of associated factors remained generally consistent. Despite these differences, core determinants such as forgetfulness, treatment burden, psychological distress, and social support were consistently observed across subgroups, suggesting the presence of common underlying mechanisms influencing adherence across diverse contexts.

## 5 Conclusion

This systematic review demonstrates that medication adherence in epilepsy is associated with a wide range of biological, psychological, treatment‐related, disease‐related, socioeconomic, cultural, and health system factors. Forgetfulness, complex medication regimens, and adverse effects were among the most frequently reported factors associated with nonadherence. Psychological factors—including depression, anxiety, negative beliefs about medications, and perceived stigma—were consistently associated with poor adherence. Social and economic challenges, particularly low income and limited access to healthcare services, were also associated with lower adherence, while family dynamics played a notable role in pediatric populations.

These findings highlight the importance of patient‐centered strategies that address both treatment‐related and psychosocial needs. Simplifying medication regimens, supporting patients with reminder tools, and providing structured education and counseling may help strengthen adherence. Future research should prioritize high‐quality longitudinal studies and standardized adherence measures to better understand these interacting factors and guide the development of targeted interventions.

## 6 Challenges and Limitations of the Study

The results of this systematic review should be considered in light of certain limitations related to the nature of the included studies and the overall scope of the review process. The majority of the included research employed cross‐sectional designs, which prevent establishing causal relationships between adherence and related factors, limiting the findings to correlational associations only. Additionally, reliance on self‐reported adherence measures such as the MMAS introduces risks of social desirability bias and recall bias, potentially leading to overestimation of adherence rates. The use of different adherence scales (e.g., MMAS‐4, MMAS‐8) across studies, conducted in various countries with diverse patient groups and methodologies, adds heterogeneity that complicates data comparison and synthesis. Finally, restricting the review to studies published only in English and Turkish increases the risk of publication bias and may have led to the exclusion of valuable unpublished research or studies published in other languages.

Moreover, during the quality assessment phase, several studies were excluded based on methodological quality as determined using the JBI Critical Appraisal Tools. These studies commonly lacked methodological clarity in areas such as sample selection, validity and reliability reporting of the measurement tools, control of confounding variables, and transparency of data collection procedures. Such methodological weaknesses contributed to a higher risk of bias and limited their suitability for inclusion in the final synthesis. To ensure transparency, the key characteristics of these excluded studies and the specific reasons for their exclusion are presented in Table S2.

## Author Contributions

**Gülcan Bahçecioğlu Turan**: conceptualization, methodology, investigation, writing – original draft, writing – review and editing, supervision. **Mizgin Demir**: conceptualization, investigation, writing – original draft, writing – review and editing, supervision.

## Funding

The authors have nothing to report.

## Conflicts of Interest

The authors declare no conflicts of interest.

## Supporting information




**Supplementary Table**: brb371550‐sup‐0001‐TableS1.xlsx

**Supplementary Table**: brb371550‐sup‐0002‐TableS2.docx

## Data Availability

All data included in this review are from previously published studies, and references are provided in the manuscript. No new datasets were generated.
