# Supplementary material for: Factors Associated With Antiseizure Medication Adherence in Patients With Epilepsy: A Systematic Review
Source: Brain Behav. 2026 Jun 14;16(6):e71550. doi: 10.1002/brb3.71550 (PMC13265823; doi:10.1002/brb3.71550)
Supplement: Supplementary file 2 — Supplementary Table: brb371550‐sup‐0002‐TableS2.docx [file BRB3-16-e71550-s002.docx]

**Supplementray Table 2.** Characteristics of Excluded Studies Due to Low JBI Critical Appraisal Score ( less than 7)

| **First Author (Year)** | **JBI Skoru** | **Reason for Exclusion** |
| --- | --- | --- |
| Loiselle (2025) | 5 | Insufficient sample size |
| Paschal (2014) | 5 | Failure to address confounding variables |
| Sweileh (2011) | 5 | Failure to address confounding variables |
| Harimanana (2013) | 6 | Insufficient sample size |
| Elsayed (2019) | 6 | Failure to address confounding variables |
| Kassahun (2018) | 6 | Insufficient sample size |
| Pant (2024) | 6 | Insufficient sample size |
| McAuley (2015) | 6 | Insufficient sample size |
| Moura (2016) | 6 | Insufficient sample size |
| Gul (2021) | 6 | Insufficient sample size |
| Henning (2019) | 6 | Insufficient sample size |
| Gündüz Oruç (2024) | 6 | Insufficient sample size |
| Bahar (2021) | 5 | Insufficient sample size |
| Aghaie & Barzegar (2024) | 6 | Insufficient sample size |
| Lee (2022) | 5 | Failure to address confounding variables |
| Shallcross (2015) | 6 | Failure to address confounding variables |
| Barot (2023) | 6 | The absence of validity/reliability information regarding measurement tools |
| Fadaye-Vatan (2017) | 6 | Inadequate sampling method and lack of mixer control |
| Carbone (2013) | 6 | Insufficient sample size |
| Govil (2021) | 6 | Insufficient sample size |
| Mohammed (2019) | 6 | Insufficient sample size |
| Lang (2023) | 6 | Insufficient sample size |
| Bedada (2020) | 6 | Insufficient sample size |
| Oo (2025) | 6 | Insufficient sample size; uncertainty regarding inclusion criteria |
